# Albendazole is effective for controlling monogenean parasites of the gills of *Piaractus brachypomus* (Serrasalmidae) and *Megaleporinus macrocephalus* (Anostomidae)

**DOI:** 10.1590/S1984-29612022057

**Published:** 2022-11-11

**Authors:** Luciano Pereira Negreiros, Eliane Xavier Souza, Tiago Araújo Lima, Marcos Tavares-Dias

**Affiliations:** 1 Instituto Federal do Acre - IFAC, Rio Branco, AC, Brasil; 2 Embrapa Amapá, Macapá, AP, Brasil

**Keywords:** Fish farm, monogenean, parasites, treatment, Piscicultura, monogenéticos, parasitos, tratamento

## Abstract

Monogenean infestations can cause high mortality in farmed fish and therefore significant economic losses. The present study investigated the efficacy of albendazole in therapeutic baths against monogeneans of *Piaractus brachypomus* Cuvier, 1818 and *Megaleporinus macrocephalus* (Garavello & Britski, 1988). For both fish, a 24 hours therapeutic bath with albendazole concentrations of 150, 300 and 500 mg/L were tested against monogeneans from the gills. The baths had an efficacy from 61.4 ± 32.9 (95%CI=64.5) against monogeneans of *P. brachypomus*, and from 95.4 ± 5.6 (95%CI=10.9) against monogeneans of *M. macrocephalus*. In *P. brachypomus,* the hepatosomatic index (HSI) in fish exposed to 150 mg/L of albendazole was higher than in fish exposed to 300 mg/L. The splenosomatic index (SSI) values in fish exposed to 150 mg/L of albendazole were lower than in fish exposed to 300 mg/L. In *M. macrocephalus*, the HSI and SSI values decreased in treatments with 150, 300 and 500 mg/L of albendazole to control and treat infestations by monogeneans. For *M. macrocephalus,* 150 mg/L of albendazole can be used to control and treat infestations by monogeneans, while for *P. brachypomus* 500 mg/L of albendazole can be used in a 24 hours bath.

## Resumo

Infestações por monogenéticos podem causar alta mortalidade entre os peixes cultivados e, portanto, perdas econômicas significativas. O presente estudo investigou a eficácia do albendazol em banhos terapêuticos contra monogenéticos de *Piaractus brachypomus* Cuvier, 1818 e *Megaleporinus macrocephalus* (Garavello & Britski, 1988). Para ambos os peixes, um banho terapêutico de 24 horas com concentrações de albendazol de 150, 300 e 500 mg/L, foi testado contra monogenéticos nas brânquias. Os banhos tiveram eficácia 61,4 ± 32,9 (IC95%=64,5) contra monogenéticos de *P. brachypomus*, e de 95,4 ± 5.6 (IC95%=10.9) contra monogenéticos de *M. macrocephalus*. Em *P. brachypomus*, o índice hepatossomático (IHS) em peixes expostos a 150 mg/L de albendazol foi maior do que em peixes expostos a 300 mg/L. Os valores do índice esplenossomático (IES) em peixes expostos a 150 mg/L de albendazol foram menores do que em peixes expostos a 300 mg/L. Em *M. macrocephalus*, os valores de IHS e IES diminuíram nos tratamentos com 150, 300 e 500 mg/L de albendazol, para controlar e tratar infestações por monogenéticos. Para *M. macrocephalus*, 150 mg/L de albendazol pode ser usado para controlar e tratar infestações por monogenéticos, enquanto para *P. brachypomus*, 500 mg/L de albendazol pode ser usado em um banho de 24 horas.

## Introduction

Over the last two decades, the global aquaculture industry has been highly successful, and continues to grow while achieving the critical objective of environmental, economic, and societal sustainability. Aquaculture is the fastest-growing food production sector in the world, and globally accounts for more fish biomass than capture fisheries, if non-edible amounts are included, and more total biomass than beef (Boyd et al., 2020; Tavares-Dias, 2021). Due to a decline in wild capture fisheries, aquaculture is a crucial component of future food security, and is essential to meet the demands of a human population expected to grow to nearly 10 billion by 2050 (Huston et al., 2020). Aquaculture is often regulated by national agencies, which represent a legal interpretation of local environmental sustainability. In Brazil, the total freshwater fish production of fish farming in 2021 was 841,005, representing an increase of 4.7% compared to 2020. In the last eight years, this production activity has grown 45%, equivalent to an average of 5.6% per year (PeixeBR, 2022). Hence, Brazil occupies 13th place in global aquaculture fish production, and is eighth in the global inland aquaculture production of finfish (Cavalli et al., 2021).

Among the native fishes that have been cultured in Brazil are *Piaractus brachypomus* Cuvier, 1818 (Serrasalmidae) and *Megaleporinus macrocephalus* (Garavello & Britski, 1988) (Anostomidae). An easy acceptance of commercial food and their fast growth have encouraged the increased production of these fish species in some regions of Brazil. Farmed in commercial fish farms, these fish are routinely stressed by intensive production management practices, leading to the emergence of parasitic diseases that naturally occur in the aquatic environment. Hence, parasitic diseases are most common in the farming of these fish species and are among the factors that strongly interfere with their growth (Martins et al., 2017; Negreiros & Tavares-Dias, 2019; Negreiros et al., 2021). Among the main taxa of disease-causing parasites in *P. brachypomus* (Negreiros & Tavares-Dias, 2019) and *M. macrocephalus* (Martins et al., 2002, 2017; Negreiros et al., 2021, 2022) are helminths monogeneans.

Monogeneans are parasitic helminths with a short and direct life cycle, the vertical transmission of which facilitates infestation levels in intensive fish farming that cause several disorders in the animal (Alves et al., 2019; Negreiros et al., 2022); such as the excessive production of mucus in the skin and gills, as well as hyperplasia, edema, the fusion of the secondary lamellae, and branchial necrosis (Tavares-Dias et al., 2021). Furthermore, secondary lesions caused by other pathogens, such as fungi and bacteria can result. Therefore, such problems show that although Brazil has an important role in the production of food from aquatic animals, the industry of fish farming yet faces many challenges.

The management and control of infestations caused by monogeneans poses a constant challenge for fish farming, as it is greatly complicated by the limited availability of licensed anthelmintic drugs, with varying degrees of effectiveness (Alves et al., 2019; Negreiros et al., 2022). In addition, the various chemical drugs that have been used against these parasites present certain problems, such as low efficacy, solubility and specificity, toxicity to host, and resistance (Santamarina et al., 1991; Tojo et al., 1992; Buchmann et al., 1992; Forwood et al., 2013; Alves et al., 2019; Tavares-Dias, 2021). Despite the broad-spectrum and tolerance of benzimidazole albendazole, to our knowledge, it has only been employed for the control and treatment of infestations caused by monogeneans in *Anguilla anguilla* Linnaeus, 1758 (Buchmann & Bjerregaard, 1990), *Onchorhynchus mykiss* (Walbaum, 1792) (Tojo et al., 1992), *Piaractus mesopotamicus* Holmberg, 1887 (Onaka et al., 2003) and *Colossoma macropomum* (Cuvier, 1816) (Alves et al., 2019). Albendazole is a versatile anthelminthic that is rapidly oxidized to its pharmacologically active metabolite, albendazole sulfoxide, after administration. Since it is widely used around the world, despite it has not yet been regulated for application in fish farming, this anthelmintic has been studied (Cordeiro et al., 2022). However, it has not been assayed to determine its efficacy in controlling monogeneans of *P. brachypomus* and *M. macrocephalus*, both fish of great economic importance to aquaculture in Brazil. Thus, the purpose of this study was to investigate the efficacy of albendazole in therapeutic baths against monogeneans of *P. brachypomus* and *M. macrocephalus*.

## Materials and Methods

### Fish and monogenean parasites

Two hundred fingerlings of *P. brachypomus* and two hundred fingerlings of *M. macrocephalus* were obtained from a commercial fish farm in Rio Branco, in the state of Acre, Brazil, and were kept at the laboratory of the Instituto Federal do Acre (IFAC), in Rio Branco (Brazil). The fish were acclimatized for 15 days in 500 L tanks with constant water flow and aeration, and were fed twice a day with commercial feed containing 35% crude protein (Guabi, Brazil). The following water parameters were maintained in the tanks: temperature at 29.1 ± 0.1 °C, dissolved oxygen at 5.6 ± 0.2 mg/L, pH at 5.4 ± 0.2, total ammonia at 0.4 ± 0.01 mg/L, alkalinity at 12.0 ± 0.1 mg/L and water hardness at 12.0 ± 0.1 mg/L. The organic matter that accumulated in the bottom of the tanks was removed once every two days. This stock of fish was used in the *in vivo* assays described below. These naturally infested fish by monogeneans were used in the experiments.

This study was developed in accordance with the principles adopted by the Brazilian College of Animal Experimentation (COBEA) and with authorization from the Ethics Committee in the Use of Animals of Embrapa Amapá (Protocol N^o^ 013- CEUA/CPAFAP).

### Anthelmintic drugs

A solution of Agebendazol® (Agener União, Brazil) with a concentration of 15% albendazole sulfoxide was used.

### Therapeutic baths against monogeneans of *P. brachypomus* and *M*. *macrocephalus*

One hundred and twenty fingerlings of *P. brachypomus* (9.0 ± 2.5 cm and 10.8 ± 9.1 g) and one hundred and twenty fingerlings *M. macrocephalus* **(**11.5 ± 3.1 cm and 33.4 ± 19.4 g), naturally parasitized by monogeneans, were randomly distributed in twelve 250 L tanks, which were kept in a static water system under constant aeration for 24 hours. The mean temperature in the tanks was 30.4 ± 0.1ºC, the dissolved oxygen content was 5.5 ± 0.2 mg/L, the pH was 5.3 ± 0.2, total ammonia was 0.5 ± 0.2 mg/L, alkalinity was 10.0 ± 0.1 mg/L, and hardness was 10.0 ± 0.1 mg/L.

One 24 hours therapeutic bath consisted of four treatments: 0, 150, 300 and 500 mg/L of albendazole (Onaka et al., 2003; Alves et al., 2019) with three replicates each, and 10 fish in each replicate, making a total of 30 fish per treatment. All treatments were performed simultaneously with the control group for each fish species. During the 24 hours bath, the behavior of the fish was observed, and they were not fed.

After the therapeutic bath with concentrations of albendazole, the fish were euthanized by medullary section, weighed (g) and measured (cm). Their gills were excised, fixed in 5% formalin and examined under a stereomicroscope to identify and quantify the monogenean parasites. The parasites were prepared for identification as recommended by Eiras et al. (2006). After parasite quantification, the prevalence and mean intensity of infection were calculated as described by Bush et al. (1997) and the efficacy of each treatment as described by Onaka et al. (2003). The monogeneans were identified according to recommendations of Cohen et al. (2013). Liver and spleen weight was measured for each fish species and used to determine the splenosomatic index (SSI) and hepatosomatic index (HSI) for each fish species (Tavares-Dias et al., 2000).

### Statistical analyses

The data were evaluated based on the Shapiro-Wilk normality test and Bartlett’s test of homoscedasticity. Because the intensity and abundance data were not normally distributed, they were analyzed by the Kruskal-Wallis test, followed by Dunn’s test for comparison among medians (Zar, 2010).

## Results

**Figure 1:**
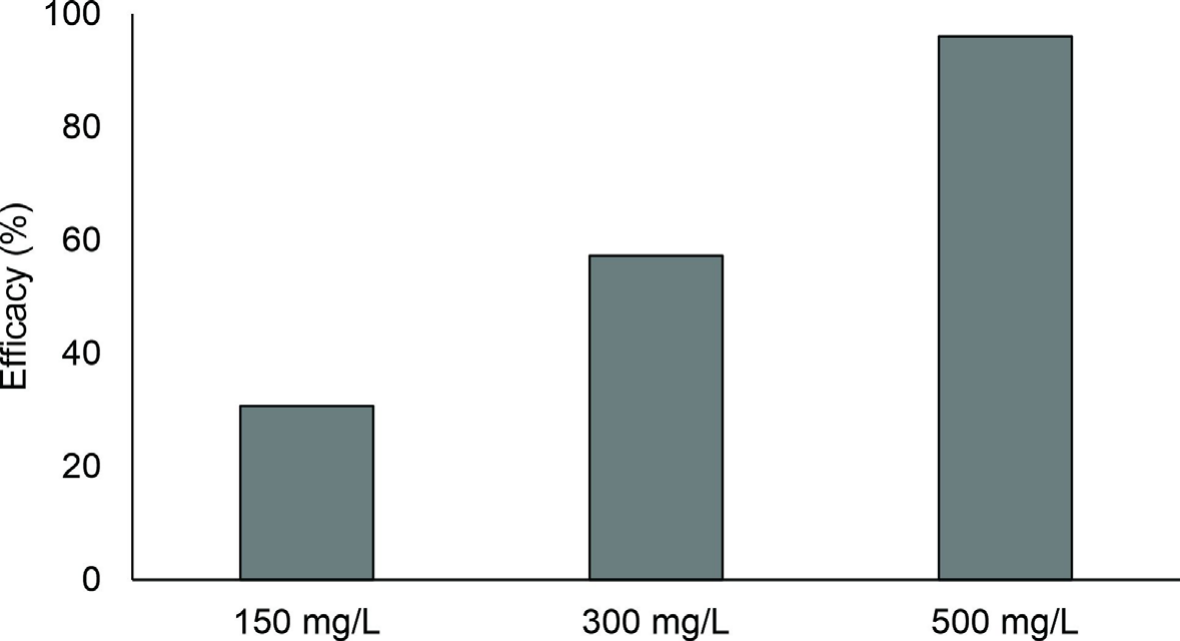
Antiparasitic efficacy of different concentrations of albendazole against monogeneans of *Piaractus brachypomus* gills.

*Piaractus brachypomus* were naturally infected by *Anacanthorus spathulatus* Kritsky, Thatcher & Kayton, 1979; *Mymarothecium viatorum* Boeger Piasecki & Sobecka, 2002 and *Anacanthorus penilabiatus* Boeger, Husak & Martins, 1995. Twenty-four hours therapeutic baths with 150, 300 and 500 mg/L of albendazole had efficacies of 30.7, 57.3 and 96.1%, respectively (Figure 1). Prevalence of monogeneans was lower in fish exposed to 500 mg/L of albendazole, while the mean intensity decreased in treatments with 150, 300 and 500 mg/L, when compared to the control fish. Hepatosomatic index values in fish exposed to 150 mg/L of albendazole were higher than in fish exposed to 300 mg/L. Splenosomatic index values in fish exposed to 150 mg/L of albendazole were lower than in fish exposed to 0 and 300 mg/L (Table 1).

**Table 1:** Infestation rates by monogeneans and body parameters of *Piaractus brachypomus* after a bath of 24 hours with different concentrations of albendazole.

Parameters	0 mg/L	150 mg/L	300 mg/L	500 mg/L
Prevalence (%)	100	100	100	43.7
Mean intensity	52.4 ± 15.6^a^	36.6 ± 15.3^b^	22.4 ± 6.2^b^	2.1 ± 3.3^c^
HSI (%)	2.2 ± 0.8^ab^	2.7 ± 1.3^b^	2.5 ± 2.7^a^	-
SSI (%)	1.1 ± 0.7^a^	0.4 ± 0.3^b^	0.8 ± 0.6^a^	-

Values express mean ± Standard deviation. Different letters in same line indicate differences by the Dunn test (p>0.05). SSI: Splenosomatic index; HIS: Hepatosomatic index.

**Figure 2:**
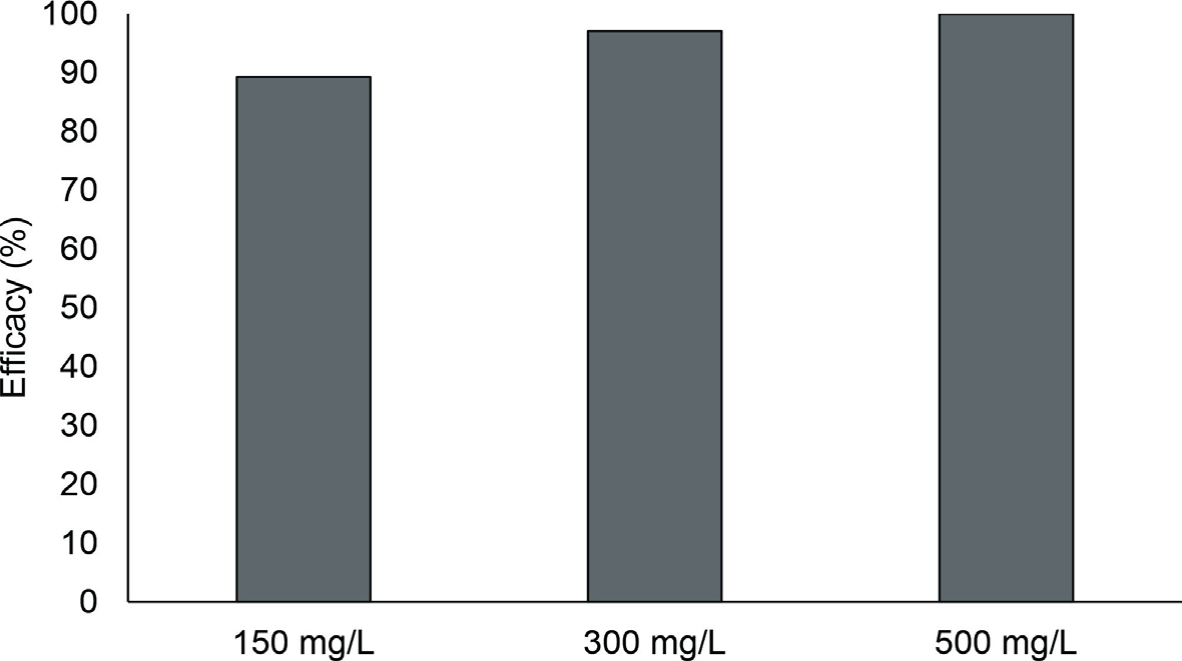
Antiparasitic efficacy of different concentrations of albendazole against monogeneans of *Megaleporinus macrocephalus* gills.

*Megaleporinus macrocephalus* were naturally infected by *Jainus leporini* Abdallah, Azevedo & Luque, 2012; *Urocleidoides paradoxus* Kritsky, Thatcher & Boeger, 1986; *Urocleidoides eremitus* Kritsky, Thatcher & Boeger, 1986 and *Tereancistrum parvus* Karling, Lopes, Takemoto & Pavanelli, 2014. Therapeutic baths of 24 hours with 150, 300 and 500 mg/L of albendazole had efficacies of 89.2, 97.1 and 100%, respectively (Figure 2). Monogenean prevalence decreased in all treatments with albendazole, as well as mean intensity. Hepatosomatic index values decreased in all treatments with albendazole, as did SSI values (Table 2).

**Table 2:** Infestation rates by monogeneans and body parameters of *Megaleporinus macrocephalus* after a bath of 24 hours with different concentrations of albendazole.

Parameters	0 mg/L	150 mg/L	300 mg/L	500 mg/L
Prevalence (%)	86.7	17.8	15.0	0
Mean intensity	11.9 ± 9.4^a^	6.2 ± 5.9^b^	2.0 ± 1.7^c^	0^d^
HSI (%)	1.3 ±0.7^a^	0.4 ± 0.1^b^	0.5 ± 0.2^b^	0.8 ± 0.9^b^
SSI (%)	1.1 ± 0.9^a^	0.2 ± 0.1^b^	0.2 ± 0.05^b^	0.5 ± 0.4^ac^
Kn	1.00 ± 1.2^a^	1.00 ± 0.02^a^	1.00 ± 0.02^a^	1.00 ± 0.07^a^

Values express mean ± Standard deviation. Different letters in same line indicate differences by the Dunn test (p>0.05). SSI: Splenosomatic index; HIS: Hepatosomatic index; Kn: Relative condition factor.

During the therapeutic baths, *P. brachypomus* and *M. macrocephalus* showed behavioral change such as agitation, but no mortality was observed.

## Discussion

Fish organ indices such as the hepatosomatic index is an indirect measure of glycogen and carbohydrate levels, and can be used to indicate the nutritional state of the fish. Splenosomatic index is a measure of both the immune status and hematopoietic capacity of the fish (Tavares-Dias et al., 2000; Voorhees et al., 2019; Negreiros et al., 2022). The differences in hepatosomatic index values between treatments with albendazole in *P. brachypomus* and *M. macrocephalus* indicate a variation in the consumption of stored energy. The differences in splenosomatic index values of *P. brachypomus* and *M. macrocephalus* indicate that hematopoietic capacity may had been affected by the treatment with albendazole.

In fish aquaculture, the drugs are administered by baths or via oral. Safe anthelmintic drugs that can be administered in therapeutic baths are suitable for use in fish farming. No concentration of albendazole caused mortality of *P. brachypomus* and *M*. *macrocephalus* or significant changes in behavior after 24 hours of exposure. Similarly, a therapeutic bath of 30 minutes with 50-500 mg/L of albendazole did not cause mortality in *P. mesopotamicus* (Onaka et al., 2003), nor in *A. anguilla* exposed to 24 hours baths with 10 and 100 mg/L of albendazole (Buchmann & Bjerregaard, 1990). However, a therapeutic bath containing 500 mg/L of albendazole caused a mortality of 6.6% of C. *macropomum* within 24 hours of exposure, but did not change the behavior of exposed fish. Therefore, these results indicate that the toxicity of albendazole is dependent on concentration and time of exposure.

Twenty-four hour therapeutic baths with 150, 300 and 500 mg/L of albendazole had efficacy of 89.2, 97.1 and 100%, respectively, against *J. leporini*, *U. paradoxus*, *U. eremitus* and *T. parvus* of *M. macrocephalus* and efficacies of 30.7, 57.3 and 96.1%, respectively, against *A. spathulatus*, *M. viatorum* and *A. penilabiatus* of *P. brachypomus*. In *A. anguilla* exposed to a 24 hours bath with 10 and 100 mg/L of albendazole, the efficacy was 100% against *Pseudodactylogyrus* sp. (Buchmann & Bjerregaard, 1990). Therapeutic baths of 30 minutes with 100, 200 and 500 mg/L of albendazole caused no mortality and had efficacies of 26.8, 52.5 and 56.9% against *A. penilabiatus* of *P. mesopotamicus* (Onaka et al., 2003). A 24 hours therapeutic bath with 500 mg/L of albendazole exhibited 48.6% antiparasitic efficacy against *A. spatulatus*, *Notozothecium janauachensis* Belmont-Jégu, Domingues & Martins, 2004; *Mymarothecium boegeri* Cohen e Kohn, 2005 and *Linguadactyloides brinkmanni* Thatcher & Kritsky, 1983 of *C. macropomum* (Alves et al., 2019). These results demonstrate differences that are interspecific of parasites, time of exposure and the concentration used in the control of monogeneans with albendazole.

Brazilian legislative framework establishes that medicinal pharmaceutical products for veterinary use must be specifically registered by MAPA, so that they can be prescribed for use in animals. In addition, to register a veterinary drug as antiparasitic, its efficacy must be greater than or equal to 90% (Cordeiro et al., 2022). Therefore, the albendazole treatments evaluated in this study achieved the required MAPA efficacy.

In conclusion, both *M. macrocephalus* and *P. brachypomus* were not sensitive to the concentrations of albendazole used. There were differences in the splenosomatic index and hepatosomatic index of *M. macrocephalus* and *P. brachypomus* after 24 hours baths with albendazole, indicating that the spleen and liver were affected by the treatment. Furthermore, it was found that 150 mg/L de albendazole can be used to control and treat infestation by monogeneans in *M. macrocephalus*, while 500 mg/L of albendazole can be used for *P. brachypomus,* in 24 hours baths.
